# Multi-stable perception balances stability and sensitivity

**DOI:** 10.3389/fncom.2013.00017

**Published:** 2013-03-20

**Authors:** Alexander Pastukhov, Pedro E. García-Rodríguez, Joachim Haenicke, Antoni Guillamon, Gustavo Deco, Jochen Braun

**Affiliations:** ^1^Center for Behavioral Brain SciencesMagdeburg, Germany; ^2^Department of Cognitive Biology, Otto-von-Guericke UniversitätMagdeburg, Germany; ^3^Centre de Recerca Matemàtica, UAB Science FacultyBarcelona, Spain; ^4^Department de Matemàtica Aplicada I, Universitat Politècnica de CatalunyaBarcelona, Spain; ^5^Institució Catalana de Recerca i Estudis AvançatsBarcelona, Spain

**Keywords:** multi-stability, binocular rivalry, adaptation, model, exploitation-exploration dilemma

## Abstract

We report that multi-stable perception operates in a consistent, dynamical regime, balancing the conflicting goals of stability and sensitivity. When a multi-stable visual display is viewed continuously, its phenomenal appearance reverses spontaneously at irregular intervals. We characterized the perceptual dynamics of individual observers in terms of four statistical measures: the distribution of dominance times (mean and variance) and the novel, subtle dependence on prior history (correlation and time-constant). The dynamics of multi-stable perception is known to reflect several stabilizing and destabilizing factors. Phenomenologically, its main aspects are captured by a simplistic computational model with competition, adaptation, and noise. We identified small parameter volumes (~3% of the possible volume) in which the model reproduced both dominance distribution and history-dependence of each observer. For 21 of 24 data sets, the identified volumes clustered tightly (~15% of the possible volume), revealing a consistent “operating regime” of multi-stable perception. The “operating regime” turned out to be marginally stable or, equivalently, near the brink of an oscillatory instability. The chance probability of the observed clustering was <0.02. To understand the functional significance of this empirical “operating regime,” we compared it to the theoretical “sweet spot” of the model. We computed this “sweet spot” as the intersection of the parameter volumes in which the model produced stable perceptual outcomes and in which it was sensitive to input modulations. Remarkably, the empirical “operating regime” proved to be largely coextensive with the theoretical “sweet spot.” This demonstrated that perceptual dynamics was not merely consistent but also functionally optimized (in that it balances stability with sensitivity). Our results imply that multi-stable perception is not a laboratory curiosity, but reflects a functional optimization of perceptual dynamics for visual inference.

## Introduction

The visual system extrapolates beyond the retinal evidence on the basis of prior experience of the visual world (Kersten et al., 2004; Hohwy et al., 2008; Friston et al., 2012). The inferential nature of vision becomes evident when prior experience shapes visual appearance (Weiss et al., 2002; Yang and Purves, 2003; Gerardin et al., 2010), in visual illusions (von Helmholtz, 1866; Bach and Poloschek, 2006; Gregory, 2009), and in visual hallucinations of certain patient populations (Ffytche et al., 2009).

The temporal dynamics of visual inferences is revealed in the phenomenon of multi-stable visual perception (von Helmholtz, 1866; Leopold and Logothetis, 1999; Blake and Logothetis, 2002; Sterzer et al., 2009). When certain ambiguous visual displays are viewed continuously, their appearance changes spontaneously from time to time. For example, some planar motion flows induce an illusory appearance of a volume moving in depth, which occasionally reverses its direction (“kinetic depth”) (Wallach and O'Connell, 1953; Sperling and Dosher, 1994). Implausible visual patterns not encountered in the natural environment induce particularly striking, multi-stable illusions. To reconcile such patterns with prior experience, even strong retinal inputs are intermittently removed from awareness, resulting in “monocular” or “binocular rivalry” (Campbell and Howell, 1972; Leopold and Logothetis, 1999; Bonneh et al., 2001; Blake and Logothetis, 2002).

Multi-stable visual perception engages a distributed network of occipital, parietal, and frontal cortical areas (Tong et al., 2006; Sterzer et al., 2009). The collective dynamics of this network reflects several stabilizing and destabilizing factors (Kohler and Wallach, 1944; Lehky, 1988; Blake et aal., 2003; Lee et al., 2007). Firstly, competition between alternative appearances stabilizes whichever appearance dominates at the time (Blake et al., 1990; Alais et al., 2010). This competition seems to be mediated by inhibitory interactions operating locally within visual representations (Lee et al., 2007; Donner et al., 2008; Maier et al., 2008). Secondly, neural adaptation of visual representations progressively weakens the dominant appearance, limiting its temporal persistence (Wolfe, 1984; Nawrot and Blake, 1989; Petersik, 2002; Blake et aal., 2003; Kang and Blake, 2010). Thirdly, neural noise initiates transitions between alternative appearances at irregular intervals (Hollins, 1980; Brascamp et al., 2006; Kim et al., 2006; Hesselmann et al., 2008; Sterzer and Rees, 2008; Sadaghiani et al., 2010; Pastukhov and Braun, 2011). Finally, volitional processes, such as attention shifts and eye movements, may also destabilize multi-stable appearance (Leopold et al., 2002; Mitchell et al., 2004; van Dam and van Ee, 2006; Zhang et al., 2011).

The interplay of stabilizing and destabilizing factors in multi-stable perception can be captured by simplistic computational models (Laing and Chow, 2002; Moldakarimov et al., 2005; Moreno-Bote et al., 2007; Noest et al., 2007; Shpiro et al., 2007; Curtu et al., 2008; Shpiro et al., 2009), at least under certain stimulus conditions (*viz*. symmetric inputs). More elaborate models are needed to reproduce multi-stable dynamics under more general conditions (Moreno-Bote et al., 2007; Wilson, 2007; Gigante et al., 2009; Seely and Chow, 2011). Here, we show that experimental observations by individual observers in particular displays tightly constrain the dynamical balance of stabilizing and destabilizing factors in multi-stable perception. Because perceptual dynamics is notoriously diverse across observers and displays (Fox and Herrmann, 1967; Borsellino et al., 1972; Walker, 1975), we expected to obtain widely disparate results. Astonishingly, we found that almost all observers operated in a narrow dynamical regime (i.e., with a particular balance of stabilizing and destabilizing factors). In addition, this “operating regime” turned out to be functionally optimal in that it balances perceptual stability and sensitivity. Our observations imply that the temporal dynamics of visual inference is functionally optimized.

## Materials and methods

### Observers

Fifteen observers (nine female, six male, including author Alexander Pastukhov) with normal or corrected-to-normal vision participated in three experiments [kinetic-depth (KD), binocular rivalry (BR) and Necker cube (NC)]. Because some observers performed multiple experiments, we obtained 24 data sets in total. The data sets from KD and BR displays were used previously to introduce the “cumulative history” measure (Pastukhov and Braun, 2011). Apart from Alexander Pastukhov, all observers were naïve to the purpose of the experiment and were paid to participate. Procedures were approved by the medical ethics board of the Otto-von-Guericke Universität, Magdeburg: “Ethikkomission der Otto-von-Guericke-Universität an der Medizinischen Fakultät.”

### Apparatus

Stimuli were generated online and displayed on a 19” CRT screen (Vision Master Pro 454, Iiyama, Nagano, Japan), with a spatial resolution of 1600 × 1200 pixels and a refresh rate of 100 Hz. The viewing distance was 95 cm, so that each pixel subtended approximately 0.011°. Background luminance was 26 cd/m^2^. Anaglyph glasses (red/cyan) were used for the dichoptic presentation.

### Multi-stable displays

**Figure 1:**
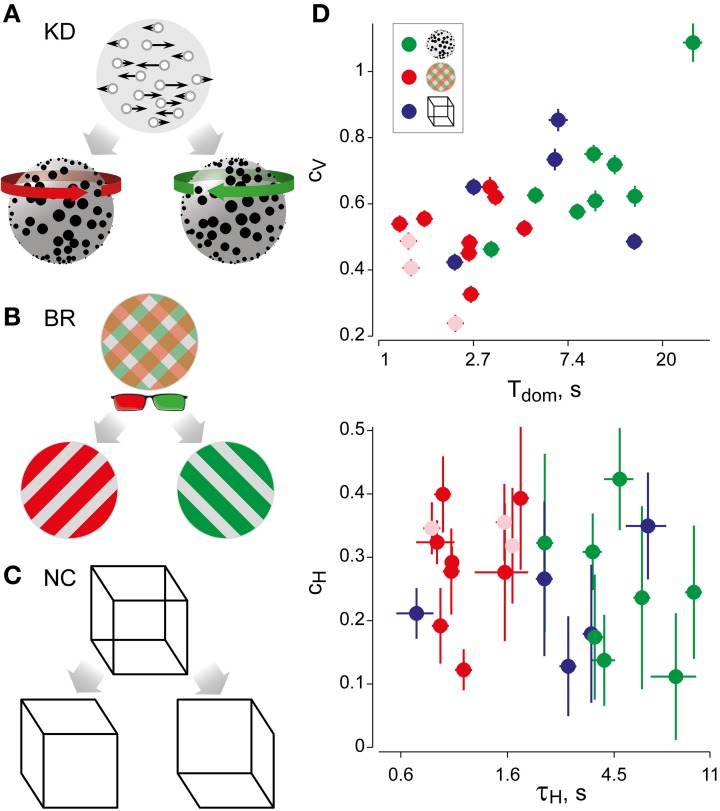
**Experimental displays and statistical measures of multi-stable dynamics. (A)** Kinetic depth (KD) display—viewing planar motion, observers perceive a volumetric rotation in either of two directions. **(B)** Binocular rivalry (BR) display—viewing different patterns with each eye (through red-green glasses), observers typically perceive either pattern. **(C)** Necker Cube (NC) display—viewing a line drawing, observers perceive one of two solid cubes. **(D)** Spontaneous perceptual dynamics varies widely between observers. Four statistical measures (mean and standard errors)—dominance duration *T*_dom_, coefficient of variation *c*_*V*_ of dominance duration, coefficient of correlation *c*_*H*_ with dominance history, time-constant τ_*H*_ of dominance history (green: 8 observers KD; red: 11 observers BR; blue: 5 observers NC). Different symbols are used for the three exceptional observers jn, lf, and np (pale symbols, see text).

The KD effect stimulus (Figure 1A) consisted of an orthographic projection of 300 dots distributed on a sphere surface (radius 3°). Each dot was a circular patch with a Gaussian luminance profile (σ = 0.057°) and a maximal luminance of 63 cd/m^2^. The sphere was centered at fixation and rotated around the vertical axis with a period of 4 s. As front and rear surface are not distinguished, the orthographic projection was perfectly ambiguous and consistent with either a clockwise or a counter-clockwise rotation around the axis. Observers perceive a three-dimensional sphere, which reverses its direction of rotation from time to time.

The BR stimulus (Figure 1B) consisted of two gratings presented dichoptically at fixation (radius, 0.9°; spatial frequency 2 cycles/degree). One grating was tilted leftward by 45° and the other rightward by 45°. The right-eye grating (green, visible only through the green filter) grating was kept at 50% contrast, while the contrast of the left-eye grating (red, visible only through the red filter) was adjusted for each subject to balance perceptual strengths. BR gives rise to several alternative perceptual states: two uniform percepts of either the left- or right-eye grating as well as different kinds of transitional percepts. Transitional percepts may be “fused” (i.e., both gratings are perceived) and/or “fragmented” (i.e., parts of both gratings are perceived in different image regions).

The NC stimulus (NC, Figure 1C) consisted of a line drawing of a 3D cube (size 3°). Observers perceive a 3D cube, which reverses its depth from time to time.

### Experimental procedure

Observers viewed the display continuously and reported the presence and identity of a clear and uniform percept. Observers pressed either the (←) key [for left rotation, left-eye (red) grating, up-and-left looking cube], or the (→) key [for right rotation, right-eye (green) grating or down-and-right looking cube], or (↓) key (for mixed or patchy percepts). Each presentation lasted for 5 min, separated by a compulsory break of (at least) 1 min. Consistent with previous reports (Lehky, 1995; Mamassian and Goutcher, 2005) reversal rates slowed during the initial part of the block, so that only the last 4 min (minus the final, incomplete dominance period) of each presentation were analyzed. Total observation time was 60 min (12 blocks) per observer for KD, 90 min (18 blocks) per observer for BR stimulus and 50 min (10 blocks) per observer for NC. Average number of clear percepts per block was 36 for KD, 110 for BR, and 45 for NC.

### Observables

The perceptual dynamics was characterized in terms of four statistical measures (see Figure 1D and Table 1), each of which varied widely between observers and displays. In addition, the distribution of dominance times was established in the form of a histogram.

**Table 1:** **Observables**.

	**Observable**	**KD**	**BR**	**NC**
*T*_dom_	Mean dominance period	11.4 ± 7.6 s	2.4 ± 1.05 s	6.6 ± 5 s
*C*_*v*_	Coefficient of variation of dominance periods	0.67 ± 0.18	0.48 ± 0.12	0.63 ± 0.17
*c*_*H*_	Linear correlation coefficient between cumulative history and subsequent dominance duration	0.24 ± 0.10	0.30 ± 0.08	0.23 ± 0.08
τ_*H*_	Exponential decay that maximizes *c*_*H*_	5.2 ± 0.85 s	1.2 ± 0.1 s	3.2 ± 0.9 s
γ_*H*_	τ_*H*_ expressed as a multiple of *T*_dom_	0.54 ± 0.21	0.56 ± 0.28	0.52 ± 0.21
	Balance between percepts	0.5 ± 0.007	0.49 ± 0.005	0.5 ± 0.021
	*p*-value for KS-test against Gamma distribution	0.74 ± 0.05	0.69 ± 0.07	0.66 ± 0.06
	*p*-value for KS-test against exponential distribution	0.09 ± 0.04	<0.001	<0.001
	*p*-value for KS-test against Gaussian distribution	0.09 ± 0.02	0.05 ± 0.01	0.17 ± 0.06

First four observables were used to constrain the model.

#### Dominance distribution

From a sequence of dominance periods *T*_*i*_ (*i* = 1,…, *N*), we computed the mean dominance time *T*_dom_ and the coefficient of variation *C*_*v*_ as

(1) $$T_{\text{dom}}=\frac{1}{N}\sum_{i=1}^{N}T_{i}$$

(2) $$C_{v}=\frac{1}{T_{\text{dom}}}\sqrt{\frac{1}{N-1}\sum_{i=1}^{N}{\left(T_{i}-T_{\text{dom}}\right)}^{2}}$$

As is typical for multi-stable percepts (Fox and Herrmann, 1967; Borsellino et al., 1972; Walker, 1975), average dominance periods varied greatly between observers and stimuli (*T*_dom_ in Table 1). In addition, dominance periods were highly variable (*C*_*v*_ in Table 1). However, the two alternative percepts dominated for comparable amounts of time (see Table 1). Patchy appearances of the BR display lasted for 1.05 ± 0.42 s.

To characterize the shape of the observed distributions of dominance times (either from human observers), we fitted the empirical distribution with a Gamma distribution with free parameters α (shape) and λ (rate)

(3) $$G(t)=\frac{1}{\Gamma (\alpha )}t^{\alpha -1}\lambda^{\alpha }e^{-\lambda t}$$

an exponential distribution with free parameter λ (rate)

(4) $$E(t)=\lambda e^{-\lambda t}$$

and a Gaussian distribution with free parameters μ (mean) and σ (variance)

(5) $$N(t)=\frac{1}{\sqrt{2\pi \sigma }}e^{-\frac{{\left(t-\mu \right)}^{2}}{2\sigma }}$$

Goodness of fit was assessed by means of KS tests. Human dominance distributions were fitted well by Gamma distributions (shape parameter α = 3.7 ± 0.7), but not by either exponential or normal distributions (Table 1), as expected from previous work (Levelt, 1967; Blake et al., 1971; Walker, 1975; Murata et al., 2003).

#### History-dependence

It is well known that successive dominance periods of the same percept tend to exhibit a marginally significant, negative correlation (van Ee, 2009; Kang and Blake, 2010), which is presumably due to neural adaptation. Recently, we have introduced a novel and more sensitive measure for this history-dependence, termed “cumulative history” (Pastukhov and Braun, 2011), which involves both a correlation coefficient, *c*_*H*_, and a characteristic time-constant, τ_*H*_ (Table 1).

The analysis of “cumulative history” in reversal sequences is described in detail by Pastukhov and Braun (2011). Briefly, the observed record of dominance reports *S*_*x*_(*t*) is convolved with a leaky integrator (Tuckwell, 2006) to compute hypothetical states *H*_*x*_(*t*) of selective neural adaptation of percept *x*:

(6) $$\begin{array}{l} \tau_{H}\frac{dH_{x}}{dt}=-H_{x}(t)+S_{x}(t)\Leftrightarrow H_{x}(t)=\frac{1}{\tau_{H}}\int_{0}^{t}S_{x}(t^{\prime })\\ \;\exp\left(-\frac{\left(t-t^{\prime }\right)}{\tau_{H}}\right)dt^{\prime }, \end{array}$$

where *x* denotes a uniform percept, τ_*H*_ is a time-constant, and *H*_*x*_(0) = 0. *S*_*x*_(*t*) takes values of 1 for dominance, 0.5 for patchy dominance (BR only), and 0 for non-dominance. The cumulative history *H*_*x*_(*t*) reflects both how long and how recently a given percept has dominated in the past. In the absence of “patchy” appearances, the cumulative histories of two competing percepts *x* and *y* sum to unity (*H*_*x*_ + *H*_*y*_ = 1).

**Figure 2:**
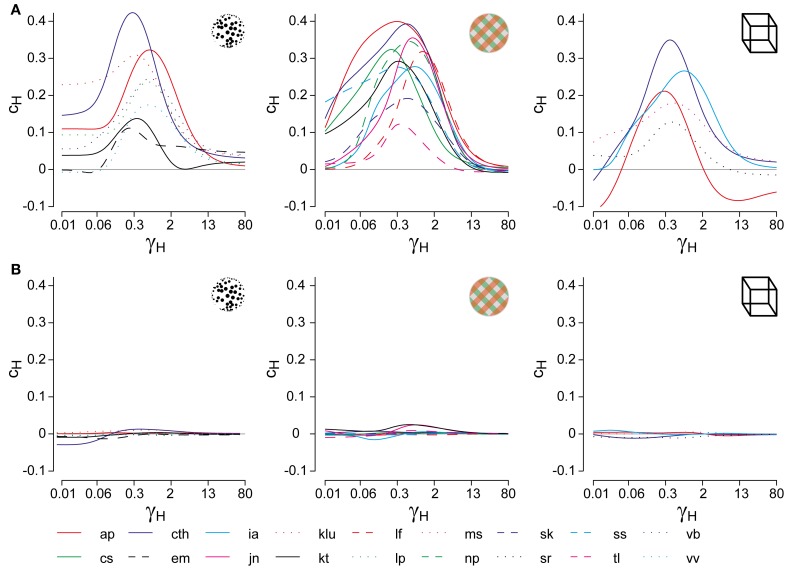
**Analysis of cumulative history in terms of *c*_*H*_ and τ_*H*_.** As described in “Materials and Methods,” correlations between cumulative history values *H*(*t*_*i*_) at reversal times *t*_*i*_ and subsequent dominance periods *T*_*i*_ were computed for different values of τ_*H*_, in order to determine the maximal value of *c*_*H*_ and its associated value of τ_*H*_. **(A)** Correlation results for all displays and observers, *c*_*H*_ as a function of τ_*H*_, where τ_*H*_ is normalized to the average dominance period *T*_dom_ of each observer (γ_*H*_ = τ_*H*_/*T*_dom_). All data sets exhibit a significant maximum, which quantifies the subtle but significant history-dependence of dominance periods in terms of *c*_*H*_ and τ_*H*_. **(B)** Analysis of shuffled reversal sequences: all dominance periods were drawn randomly and with replacement from the observed distribution of dominance periods. No significant correlations (indications of history-dependence) remain after shuffling. Panel **(A)** is modified from Figure 3 of Pastukhov and Braun (2011).

For suitable values of τ_*H*_, the cumulative history *H*(*t*) at a reversal time *t* correlates significantly with the subsequent dominance period *T*_*i*_. Specifically, if *t*_*i*_ marks the beginning of dominance period *T*^*i*^_*x*_, we computed linear correlations between *H*_*x*_(*t*_*i*_) and ln(*T*^*i*^_*x*_) for all four possible combinations of history and percept (*H*_*x*_ × *T*_*x*_, *H*_*x*_ × *T*_*y*_, *H*_*y*_ × *T*_*y*_, and *H*_*y*_ × *T*_*x*_). The average absolute correlation was obtained for values of τ_*H*_ ranging from 0.01 to 60 s, in order to determine the maximal correlation coefficient *c*_*H*_ and its associated value of τ_*H*_ (Figure 2A).

To verify that the values of *c*_*H*_ and τ_*H*_ represented a true history-dependence (and not just the spectral characteristics of the data), we repeated the analysis with shuffled reversal sequences (dominance times drawn randomly with replacement from the observed distribution). No significant correlations *c*_*H*_ were observed in the shuffled data sets (Figure 2B).

### Computational modeling

To generate a wide variety of dynamical regimes, we simplified the rate model of Laing and Chow (see Laing and Chow, 2002), which has been analyzed and extended by several other groups (Moreno-Bote et al., 2007; Shpiro et al., 2007; Curtu et al., 2008; Shpiro et al., 2009). Two neural populations represent competing percepts. Each population excites itself and inhibits the other population. In addition, each population is subject to adaptation in the form of a threshold elevation and to stochastic effects in the form of additive noise:

(7) $$\tau_{r}{\dot{r}}_{1,2}=-r_{1,2}+F\left(\alpha r_{1,2}-\beta r_{2,1}-\phi_{a}a_{1,2}+I_{1,2}+n_{1,2}\right)$$

(8) $$\tau_{a}{\dot{a}}_{1,2}=-a_{1,2}+r_{1,2}$$

where *r*_1, 2_ is population activity, *a*_1, 2_ is adaptive state, *I*_1, 2_ = *I*_0_ is the strength of the (common) input to both populations, and *n*_1, 2_ is colored noise. The sigmoidal function *F*(*x*) is defined as

(9) $$F(x)=\frac{1}{1+\exp\left(-\frac{x}{k}\right)}$$

The parameters α and β control, respectively, the self-excitation and mutual inhibition of the two populations. In a sense, they represent the influence of prior experience. We set α = 0 because we were not interested in the regime of self-sustaining activity. The parameter ϕ_*a*_ sets the strength of neural adaptation and *I*_1, 2_ represents current retinal input. We typically set *I*_1_ = *I*_2_ = *I*_0_. The parameters τ_*r*_ and τ_*a*_ are the characteristic time-constants of activity and adaptive state, respectively. Finally, additive noise *n*_1, 2_ is provided by two independent Ornstein–Uhlenbeck processes with variance σ_*n*_ and time-constant τ_*n*_:

(10) $${\dot{n}}_{i}=-\frac{n_{i}}{\tau_{n}}+\sqrt{\frac{2\sigma_{n}^{2}}{\tau_{n}}}\xi_{i}$$

from two independent sources of Gaussian noise ξ_1, 2_ with

(11) $$\langle \xi_{i}(t)\xi_{i}(t+\epsilon )\rangle =\delta (\epsilon ),\;\langle \xi_{i}\rangle =0$$

Thus, the signal-to-noise ratio of the retinal input is given by *I*_1, 2_/σ_*n*_. To predict perceptual dominance *S*_*x*_(*t*), we assume a reversal to percept *x* whenever the associated activity *r*_*x*_ is 25% larger than the activity associated with the other percept.

### Model parameters

The parameters τ_*r*_, τ_*n*_, and *k* remained fixed at τ_*r*_ = 10 ms, τ_*n*_ = 100 ms, and *k* = 0.1. The dynamical regime (stationary, oscillatory, or bistable) depends largely on three parameters, with *I*_0_ setting the general activity and overall stability of percepts, β the strength of mutual inhibition, and ϕ_*a*_ the strength of adaptation. This three-dimensional parameter space was explored in the limits of *I*_0_ ∈ [0, 2], β ∈ [0, 2], and ϕ_*a*_ ∈ [0, 1]. For every given triplet of *I*_0_, β, and ϕ_*a*_ values, we additionally simulated all combinations of τ_*a*_ ∈ [1.0, 1.2, 1.4, 1.6, 1.8, 2.0, 3.0, 4.0, 5.0, 6.5, 8.0] s and σ_*n*_ ∈ [0.01, 0.03, 0.05, …, 0.35]. The latter two parameters influence *T*_dom_ and *C*_*v*_, but are inconsequential for the dynamical regime.

For convenience, all model parameters and associated value ranges are listed here: α = 0, β ∈ [0, 2], ϕ_*a*_ ∈ [0, 2], *I*_1, 2_ = *I*_0_ ∈ [0, 2], σ_*n*_ ∈ [0.01, 0.35], τ_*a*_ ∈ [1, 8] s, τ_r_ = 10 ms, τ_*n*_ = 100 ms, *k* = 0.1.

### Simulations

To generate multi-stable dynamics and to predict psychophysical observables, three simulations of 500 s each were performed for every combination of model parameters. If the value of any predicted observable varied too much (*C*_*v*_ > 0.5), five simulations of 3000 s were performed. The values of predicted observables were then compared with the empirical values of *T*_dom_, *C*_*v*_, τ_*H*_, and *c*_*H*_ for each observer and display. If all four predictions fell within 25% of the empirical values, the corresponding combination of model parameters *I*_0_, β, and ϕ_*a*_ was marked as a “match.” Typically, a match was obtained for σ_*n*_ ≈ 0.15.

### Frequency resonance simulations

To investigate frequency resonance, the two inputs were modulated in anti-phase with different periods *T*_*s*_

(12) $$I_{1,2}=I_{0}\pm \Delta I\cos\left(\frac{2\pi t}{T_{s}}\right)$$

and the distribution of dominance periods *P*_res_(*T*) was determined for different values of *T*_*s*_ (Δ*I* = 0.2*I*_0_). As shown in Figure 12A, this distribution exhibits resonance peaks at odd multiples of the half-period of modulation $\frac{T_{s}}{2}$. The most pronounced resonance typically occurs for *HP* = *T*_*s*_/2 = *T*_dom_.

To compare frequency resonance at different points in the three-dimensional parameter space *I*_0_ ∈ {0, 2}, β ∈ {0, 2}, and ϕ_*a*_ ∈ {0, 1}, two simulations of 4000 s were performed at each point with medium noise σ_*n*_ = 0.15 and τ_*a*_ = 1 s. One simulation established the unperturbed distribution of dominance periods *P*_ref_(*T*) and the mean dominance time 〈*T*_dom_〉. In the other simulation, inputs *I*_1, 2_ were modulated in anti-phase at the best resonance frequency *T*_*s*_ = 2〈*T*_dom_〉 and the distorted distribution of dominance periods *P*_res_(*T*) was established.

The resonance coefficient *P*_1_ was then computed as

(13) $$P_{1}=\left[\int_{\frac{HP}{2}}^{\frac{3\;HP}{2}}P_{\text{res}}(T)dT\right]{\left[\int_{\frac{HP}{2}}^{\frac{3HP}{2}}P_{\text{ref}}(T)dT\right]}^{-1}$$

where *HP* = *T*_*s*_/2.

Finally, to localize the bifurcation surfaces, simulations of 600 s were performed throughout the three-dimensional parameter space in the absence of noise (σ_*n*_ = 0, τ_*a*_ = 1 s). Starting from an asymmetric initial condition (*r*_1,2_ = *a*_1,2_ = [0, 1]), we determined whether activities migrated to identical steady-state values *r*_1_ = *r*_2_ = *a* (stationary regime), periodically reversed in rank order to exhibit values with *r*_1_ < *r*_2_ (oscillatory regime), or migrated to steady-state values with the same rank order *r*_1_ > *r*_*2*_ (bistable regime).

### Simulation equipment

Simulations were performed on a Linux cluster (Suse Linux Enterprise Server 10, Matlab R2007a, C++ compiler gcc 20070115) with five nodes (each with four processors Intel(R) Xeon(R) CPU E5430 @ 2.66 GHz and 8 GB RAM).

## Results

We studied three canonical multi-stable displays (Figures 1A–C and Video S1): KD in a two-dimensional projection of a rotating cloud of dots (Wallach and O'Connell, 1953), BR between two gratings of different color and orientation (Wheatstone, 1838; Meng and Tong, 2004), and the NC (Necker, 1832). Observers viewed each display continuously for 5 min and reported its appearance either as rotating in depth “front left” or “front right” (KD), or as “uniformly red,” “uniformly green,” or “patchy” (BR), or as the marked corner pointing to “front” or “back” (NC display).

### Dominance distribution and history-dependence

For each observer and display, we characterized perceptual dynamics in terms of several statistical measures (Figure 1D and Table 1). The distribution of dominance times was binned into a histogram and summarized in terms of mean dominance duration, *T*_dom_, and coefficients of variation, *C*_*v*_. Both dominance durations (1–22 s) and coefficients of variation (0.2–1.1) varied widely between observers and displays, as is typical for multi-stable percepts (Fox and Herrmann, 1967; Borsellino et al., 1972; Walker, 1975). Also as expected (Levelt, 1967; Blake et al., 1971; Walker, 1975; Murata et al., 2003), the distributions of dominance times resembled Gamma functions with a comparatively narrow range of shape parameters α (3.7 ± 0.6). Specifically, the empirical distributions were consistently fit better by a Gamma distribution (KS-test *p* = 0.7 ± 0.06), than by either an exponential distribution (*p* = 0.03 ± 0.02) or a Gaussian distribution (*p* = 0.09 ± 0.03).

In addition, we captured the subtle history-dependence of dominance times in terms of a correlation coefficient, *c*_*H*_, and a characteristic time-constant, τ_*H*_ (Figures 1D, 2). Due to the destabilizing effect of neural adaptation, successive periods dominated by the same appearance often exhibit a marginally significant, negative correlation (van Ee, 2009; Kang and Blake, 2010; Pastukhov and Braun, 2011). Recently, we have introduced a more sensitive, integral measure, dubbed “cumulative history,” of how long and how recently a given percept has dominated in the past (Hudak et al., 2011; Pastukhov and Braun, 2011). This measure reveals that individual dominance periods are consistently and significantly influenced by prior perceptual history (see “Materials and Methods” and Figure 2). For different observers and displays, the values of *c*_*H*_ ranged from 0.1 to 0.4 and the values of τ_*H*_ from 0.6 to 10 s, quantifying the history-dependence in each case (Table 1). Our use of this “cumulative history” measure constitutes an important difference to earlier work (Shpiro et al., 2009).

### Dynamical regimes of LC-model

**Figure 3:**
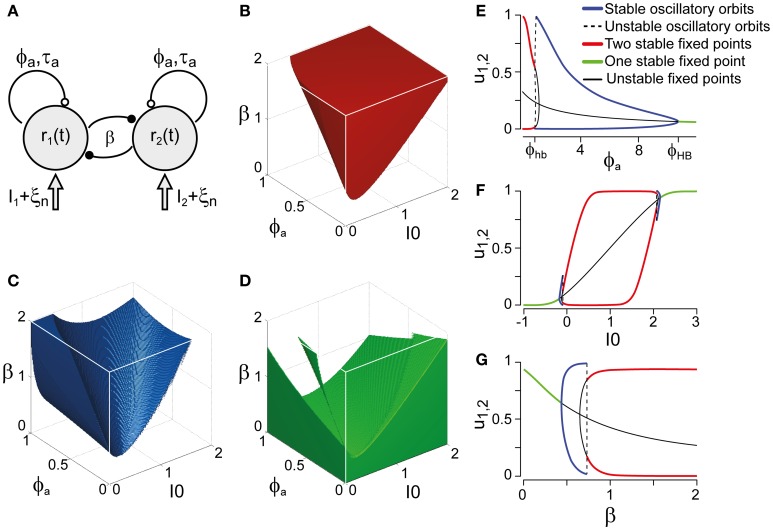
**Bifurcation analysis of a class of generative models. (A)** Generative models (schematic) for multi-stable dynamics with two neural populations (after Laing and Chow, 2002). Population activities *r*_1,2_, strength of cross-inhibition β, visual input *I*_1,2_ = *I*_0_, strength of neural adaptation ϕ_*a*_, time-constant τ_*a*_ of neural adaptation, independent neural noise ξ_*n*_. Dynamical regimes depend largely on only three parameters: β, ϕ_*a*_, and *I*_0_. **(B)** Bistable region (red volume and red lines on bifurcation diagrams EFG), see also Figure 4A. Without neural noise, activities *r*_1,2_ approach one of two steady-states with disparate activity levels (one high, one low). With noise, transitions between the two steady-states occur at irregular intervals. **(C)** Oscillatory regime (blue volume and blue lines on bifurcation diagrams EFG), see also Figure 4B. Without noise, activities *r*_1,2_ oscillate in counter-phase between low and high levels. Neural noise renders the alternation more irregular. **(D)** Stationary regime (green and green lines on bifurcation diagrams EFG). Activities *r*_1,2_ approach a single steady-state, with or without noise. **(E–G)** Bifurcation analysis of parameters ϕ_*a*_, *I*_0_, and β. **(E)** Dependence on ϕ_*a*_, revealing bistable, oscillatory, and stationary regimes (β = 1.75, *I*_0_ = 0.5). Hopf bifurcations are marked ϕ_hb_ and ϕ_HB_. **(F)** Dependence on *I*_0_, showing a central bistable regime flanked by oscillatory and stationary regimes on either side (β = 1.75, ϕ_*a*_ = 0.25). **(G)** Dependence on β, showing bistable, oscillatory, and stationary regimes (ϕ_*a*_ = 0.25, *I*_0_ = 0.5).

Next, we compared our perceptual observations to a class of generative models for multi-stable dynamics. We chose the model formulated by Laing and Chow (2002) and investigated by several other groups (Moldakarimov et al., 2005; Moreno-Bote et al., 2007; Noest et al., 2007; Shpiro et al., 2007; Curtu et al., 2008; Shpiro et al., 2009), which strikes a dynamical balance between competition β, adaptation ϕ_*a*_, and input strength *I*_0_ (Figure 3). Depending on this balance, the “LC-model” is able to generate sequences of perceptual reversals with a wide range of dominance distributions and history-dependencies. Note that all models incorporating adaptation, such as (Laing and Chow, 2002; Moldakarimov et al., 2005; Moreno-Bote et al., 2007; Noest et al., 2007; Shpiro et al., 2007; Curtu et al., 2008; Shpiro et al., 2009), necessarily predict a degree of history-dependence.

Whereas the LC-model generates a continuum of possible dynamics, one may technically distinguish two regimes: a *bistable* or fluctuation-driven regime in which adaptation ϕ_*a*_ is weak [ϕ_*a*_ < ϕ^*hb*^_*a*_(β, *I*_0_)] and dominance periods are terminated by noise (Figure 3B), and an *oscillatory* or limit-cycle regime in which adaptation ϕ_*a*_ is strong enough [ϕ_*a*_ > ϕ^*hb*^_*a*_(β, *I*_0_)] to terminate each dominance period on its own (Figure 3C). The *stationary* regime of the model does not generate reversals and is not relevant here (Figure 3D).

**Figure 4:**
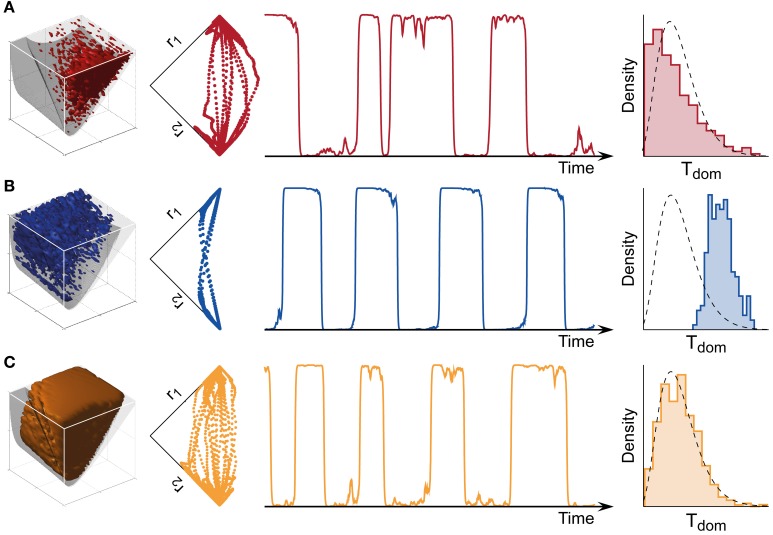
**Bistable, oscillatory, and intermediate dynamics. (A)** Bistable dynamics obtained deeply within the bistable regime (far left, cf. Figure 3B). Driven largely by noise, it is characterized by irregular trajectories in state space (middle left), aperiodic dominance reversals (middle right), and an approximately exponential distribution of dominance times (far right). **(B)** Oscillatory dynamics obtained deeply within the oscillatory regime (far left, cf. Figure 3C). Driven largely by adaptation, it is characterized by regular trajectories in state space (middle left), periodic dominance reversals (middle right), and an approximately Gaussian distribution of dominance times (far right). **(C)** The multi-stable dynamics of human observers falls between these two extremes: it exhibits irregular trajectories (middle left), aperiodic reversals (middle right), and a Gamma-like distribution of dominance times (far right). With suitable levels of noise, a large parameter volume (far left) can result in realistic (human-like) distributions of dominance times (see text for details).

Both the *bistable* and the *oscillatory* regimes of this model generate multi-stable dynamics, but with important differences in detail (Figure 4). A typical *bistable* dynamics is dominated by noise, resulting in irregular trajectories through state space, aperiodic dominance reversals, and an approximately exponential distribution of dominance times (Figure 4A). In marked contrast, a typical *oscillatory* dynamics is dominated by adaptation, with state-space trajectories describing a stereotypical limit-cycle, periodic dominance reversals, and an approximately Gaussian distribution of dominance times (Figure 4B).

The perceptual dynamics of human observers tends to fall between these two extremes. Typically, human dominance periods exhibit a Gamma distribution with shape factor α between 3 and 4 (Murata et al., 2003), a distribution shape that is intermediate between exponential and Gaussian distributions Figure 4C). On this basis, it has been suggested that the operating regime of human multi-stable perception may lie near the boundary between *bistable* and *oscillatory* regimes (Shpiro et al., 2009).

### Realistic dominance distribution

We will now show that the distribution shape of dominance periods does not usefully constrain the dynamical regime of multi-stable perception. In essence, this is because the LC-model is highly redundant in the sense that many combinations of parameters generate equally realistic (Gamma-like) distribution shapes. To establish this point, we carried out extensive simulations, independently varying competition β ∈ [0, 2], adaptation ϕ_*a*_ ∈ [0, 1], input strength *I*_0_ ∈ [0, 2], noise amplitude σ_*n*_ ∈ [0.01, 0.35], and adaptation time-scale τ_*a*_ ∈ [1 s, 8 s]). For each parameter combination (β, ϕ_*a*_, *I*_0_, σ_*n*_, τ_*a*_), we generated reversal sequences and established the best-fitting Gamma, exponential, and Gaussian functions for the resulting distribution of dominance times.

The dominance distribution generated by a parameter combination (β, ϕ_a, *I*_0_, σ_*n*_, τ_*a*_) was classified as realistic or human-like, if it was well fit by a Gamma distribution with shape parameter α ∈ [3.1, 4.3] (KS-test *p* > 0.7) and less well by either exponential and Gaussian distributions. The parameter volume in which the LC-model generated human-like distributions of dominance times is shown in Figure 4C (far left). Note that the illustration shows only three of the five parameters. Only some, not all, choices of the two hidden parameters σ_*n*_, τ_*a*_ resulted in realistic distributions. The depicted volume encompassed approximately 57% of the possible volume and was not restricted to the boundary between *bistable* and *oscillatory* regimes.

Accordingly, the distribution shape of dominance periods, taken by itself, does not usefully constrain the dynamical regime of multi-stable perception, as has been claimed (Shpiro et al., 2009). The reason for this discrepancy is that we explored a larger range of hidden parameters σ_*n*_, τ_*a*_ than (Shpiro et al., 2009). Essentially, a realistic distribution shape can almost always be obtained if a suitable noise level σ_*n*_ and adaptation time-constant τ_*a*_ are chosen.

### Realistic dominance distribution and history-dependence

Fortunately, a far more informative set of constraints becomes available when both the dominance distribution and the history-dependence of human observers are taken into account. Comparing simulated and human perceptual dynamics, parameter combinations (β, ϕ_*a*_, *I*_0_, σ_*n*_, τ_*a*_) were considered a “match” if their statistics (*T*_dom_, *C*_*v*_, *c*_*H*_, τ_*H*_) fell within 25% of the statistics of a particular observer/display combination. In this case, we refrained from comparing distribution shapes explicitly, as this would have complicated the interpretation of the results, but would not have further constrained the parameter volumes.

**Figure 5:**
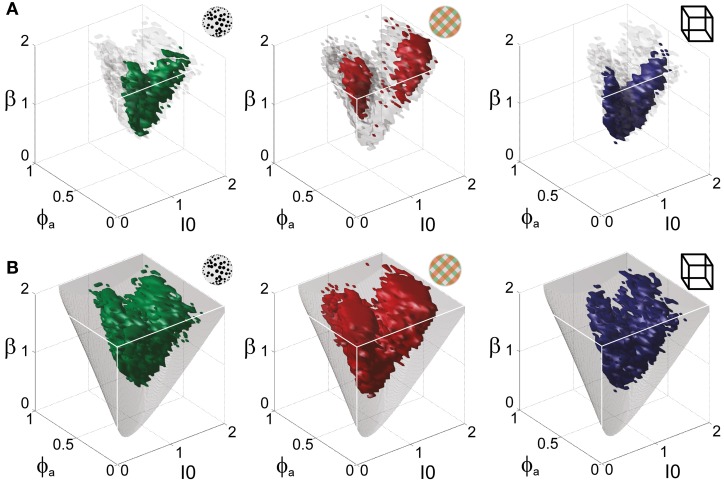
**Operating regime of multi-stable perception.** KD display (left), BR display (middle), and NC display (right). **(A)** Parameter volumes (green, red, blue) matching the perceptual dynamics of three representative human observers (*lp*, *kt*, and *ia*, respectively) in terms of both the distribution (*T*_dom_, *C*_*v*_) and the subtle history-dependence (*c*_*H*_, τ_*H*_) of dominance times. The depicted volumes fill approximately 6% of the possible volume and are here compared to the union of observers (transparent gray volumes). **(B)** Union of the matching volumes (green, red, blue) from 8, 8, and 5 observers, respectively. The matching volumes lie entirely within the bistable regime (transparent gray volumes) and fill approximately 15% of the possible volume.

**Figure 6:**
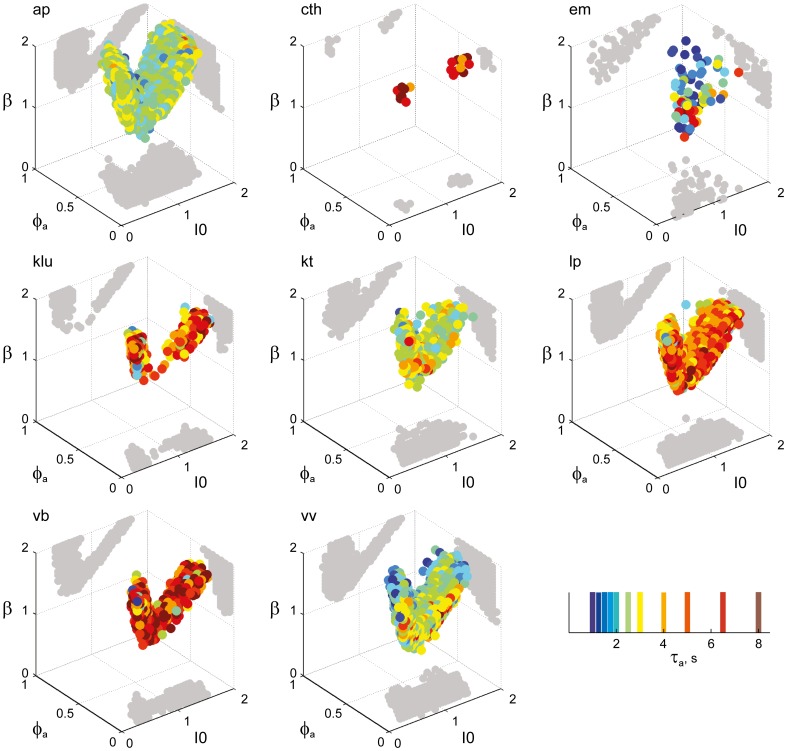
**Parameter volumes matching the perceptual dynamics of individual observers for KD displays.** For each parameter triplet *I*_0_, ϕ_*a*_, and β, different combinations of noise level and adaptation time-constant were explored in the ranges σ_n_ ∈ [0.01, 0.35] and τ_*a*_ ∈ [1 ms, 8 ms]. A “match” was declared when the statistics of synthetic reversal sequences fell within 25% of the mean values of each of the four observables 〈*T*_dom_〉, *C*_*v*_, *c*_*H*_, and τ_*H*_. The color coding indicates the value of τ_*a*_ at which each parameter triplet *I*_0_, ϕ_*a*_, and β best matched observer dynamics. For each matching volume, three orthogonal projections on different planes are shown in gray. The green volume shown on the left of Figure 5B represents the union of the volumes illustrated here.

**Figure 7:**
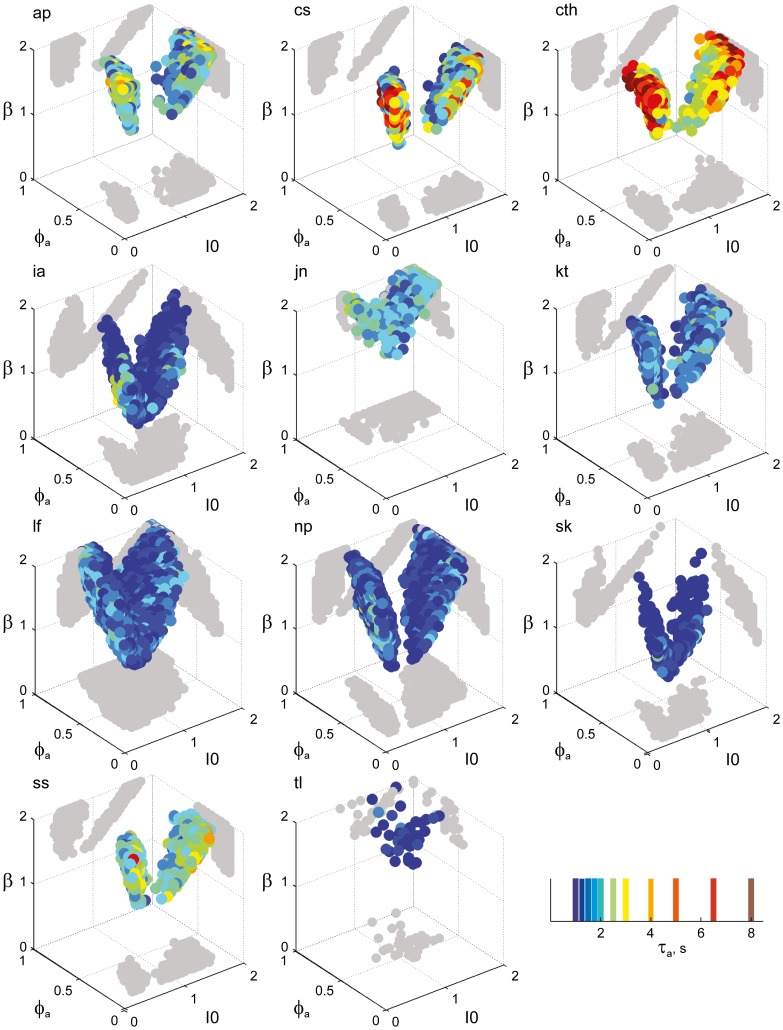
**Parameter volumes matching the perceptual dynamics of individual observers for BR displays (see Figure 6 for details).** The color coding indicates the value of τ_*a*_ at which each parameter triplet *I*_0_, ϕ_*a*_, and β best matched observer dynamics. For exceptional observers (jn, lf, and np) parameter volumes lie partially outside the stable and sensitive volume. For each matching volume, three orthogonal projections on different planes are shown in gray. The red volume shown in the middle of Figure 5B represents the union of the volumes illustrated here.

**Figure 8:**
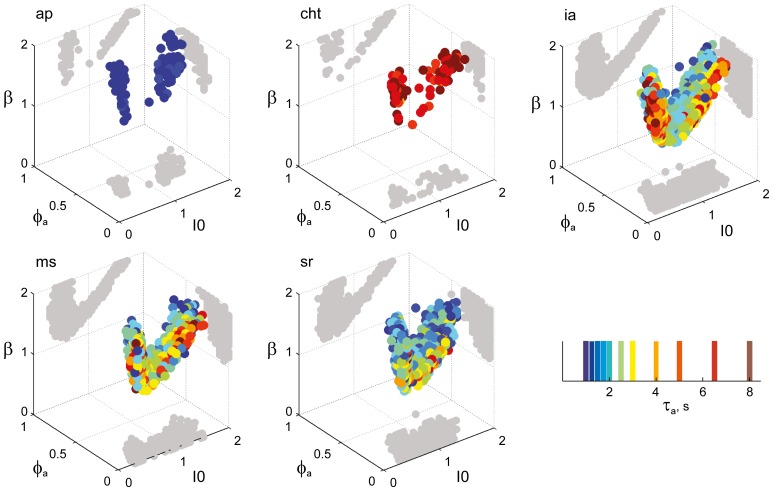
**Parameter volumes matching the perceptual dynamics of individual observers for NC displays (see Figure 6 for details).** The color coding indicates the value of τ_*a*_ at which each parameter triplet *I*_0_, ϕ_*a*_, and β best matched observer dynamics. For each matching volume, three orthogonal projections on different planes are shown in gray. The blue volume shown on the right of Figure 5B represents the union of the volumes illustrated here.

Astonishingly, the parameter combinations that matched almost all observers/displays clustered in a consistent “operating regime” of approximately 15% of the possible volume (Figure 5B): 8/8 observers of the KD display were matched by 10%, 8/11 observers of the BR display by 13%, and 5/5 observers of the NC display by 7% of the possible parameter volume. The individual results for all observers are presented in Figures 6–8. In most cases, a comparatively small and well-defined parameter volume reproduced all four statistical measures (*T*_dom_, *C*_*v*_, *c*_*H*_, τ_*H*_) (see Figure 5A for representative examples). On average, the matching volumes comprised 2.4 ± 1.1% (KD display), 4.5 ± 0.7% (BR display), and 2.9 ± 1.0% (NC display), of the possible parameter spaces (bistable and oscillatory regimes).

At this juncture, the reader may well wonder how these results depend on the 25% criterion used to define a “match” between simulated and human reversal statistics. In fact, the “envelope” of the matching volumes described above is largely independent of this criterion choice. If the parameter space (β, ϕ_*a*_, *I*_0_, σ_*n*_, τ_*a*_) is sampled at a sufficiently densely spaced points, any set of observed statistical measures (*T*_dom_, *C*_*v*_, *c*_*H*_, τ_*H*_) can be reproduced with arbitrary precision. In other words, the density of parameter sampling determines the precision with which observed statistical measures can be reproduced. The 25% criterion was chosen to obtain cohesive “matching” volumes, given the sampling grid of our simulations. For this criterion value, an observed statistics was typically reproduced by several adjacent grid locations. When a stricter criterion was used, an observed statistics tended to be reproduced only by isolated grid locations, resulting in non-cohesive or “patchy” matching volumes. In sum, the criterion choice merely affected the internal cohesiveness, but not the “envelope,” of the parameter volumes reproducing human reversal statistics.

Why should the four statistical measures (*T*_dom_, *C*_*v*_, *c*_*H*_, τ_*H*_) offer a more informative set of constraints than the shape of the dominance distribution alone? In the LC-model, distribution shape (*T*_dom_, *C*_*v*_, and higher moments) is determined by the relative strength of adaptation and noise. Accordingly, many parameter combinations produce realistic distribution shapes, provided a suitable level of noise is chosen in each case. History-dependence (*c*_*H*_, τ_*H*_), on the other hand, is less sensitive to the level of noise and therefore more informative about the absolute strength of adaptation. Thus, distribution shape and history-dependence provide largely independent constraints. That this is indeed the case was evident from the disparate parameter volumes which reproduce different sets of constraints: whereas comparatively small volumes (3.3 ± 1.6% of the possible volume) reproduced both dominance distribution (*T*_dom_, *C*_*v*_) and history-dependence (*c*_*H*_, τ_*H*_) of individual observers/displays, far larger volumes reproduced either one of these constraints (29 ± 15% for *T*_dom_, *C*_*v*_ and 44 ± 7% for *c*_*H*_, τ_*H*_).

### A consistent human “operating regime”

Overall, the multi-stable dynamics of 21/24 data sets was matched by a consistent “operating regime,” lying entirely within the *bistable* domain of the model and comprising approximately 15% of the possible volume (Figure 5B). The results from individual observers are detailed in Figure 6 (KD displays), Figure 7 (BR displays), and Figure 8 (NC displays). Only three observers of the BR display (*jn*, *lf*, *np*) exhibited an exceptional dynamics in that their brief dominance times *T*_dom_ and strong history-dependence *c*_*H*_ were matched not only in the *bistable* but also in the *oscillatory* regime of the LC-model (Figure 7).

**Figure 9:**
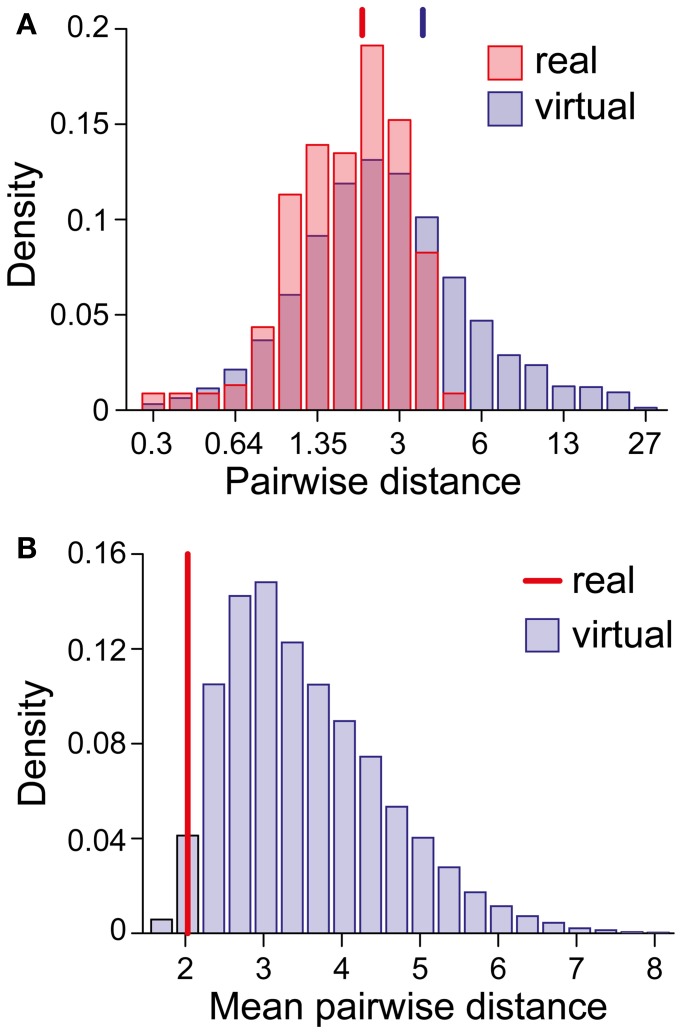
**Clustering of matching regions in (*I*_0_, ϕ_*a*_, β)-space. (A)** Distribution of center-to-center distances between the matching volumes of observer pairs (real and virtual). Vertical lines mark the distribution means. **(B)** Distribution of the *mean* of all center-to-center distances among groups of 21 virtual observers (computed over 10,000 randomly chosen sets). The vertical line (red) marks the value obtained for the 21 real observers/data sets. The likelihood that equal numbers of virtual observers cluster as tightly as real observers was <0.02.

We were astonished by this clustering, especially in view of the superficial diversity in the perceptual dynamics exhibited by different observers/displays (Figure 1D). To assess the likelihood of an accidental clustering, we shuffled the pairs of statistical measures (*T*_dom_, *C*_*v*_) and (*c*_*H*_, τ_*H*_), drawing observables randomly from the value pairs produced by real observers and recombining them to form “virtual” observers. In general, the matching volumes of these “virtual” observers were far more widely scattered (51% of the possible volume) than those of “real” observers. To quantify this further, we computed the centers of all matching volumes (mean parameter vectors) and the norms of the distances between all volume pairs. Whereas the average pair-distance was comparable for real and for “virtual” observers (2.0 ± 1.2 and 3.4 ± 3.8, respectively, Figure 9A), the group-mean for real observers was much smaller than the group-mean for equal numbers of “virtual observers” (Figure 9B), demonstrating that real observers clustered tightly in a consistent “operating regime.” The likelihood of obtaining by chance the clustering exhibited by real observers was not significant (*p* < 0.02).

### Shape and location of “operating regime”

**Figure 10:**
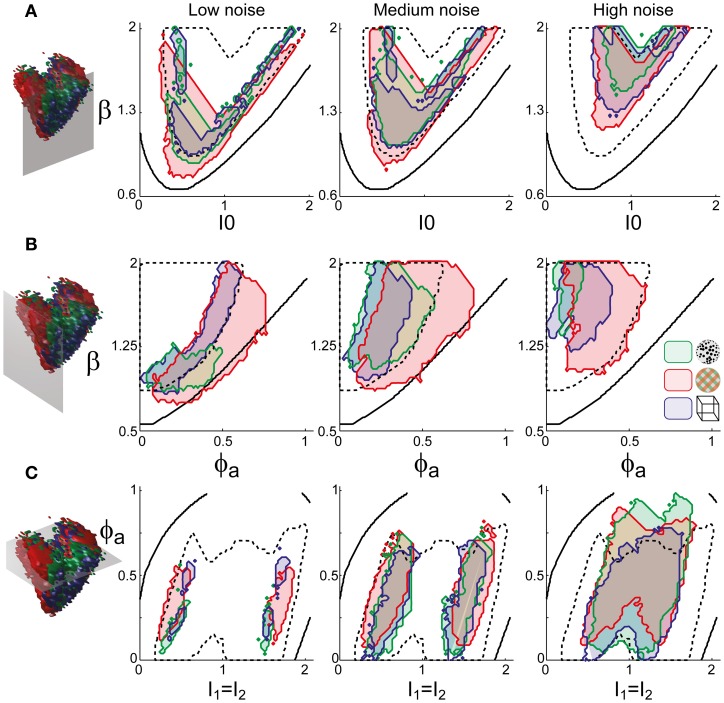
**Operating regimes of multi-stable perception for different levels of noise (planar subspaces).** The left inset relates the selected subspaces to the three-dimensional volumes of Figure 5. Several regions matching human observer dynamics with different displays and under different noise assumptions are illustrated. Specifically, the union of the matching regions of individual observers is outlined in a different color for each display (KD, BR, NC, see inset). Also marked are the bifurcation surface (black contour) and the functional “sweet spot” for medium noise (dotted black outline, see Figure 12C). Matching regions occupy different shell-like volumes, depending on the assumed level of noise (low, medium, or high). Distance to the bifurcation increases with noise. **(A)** Planar subspace ϕ_*a*_ = 0.25. **(B)** Planar subspace *I*_0_ = 0.55. **(C)** Planar subspace β = 1.71.

**Figure 11:**
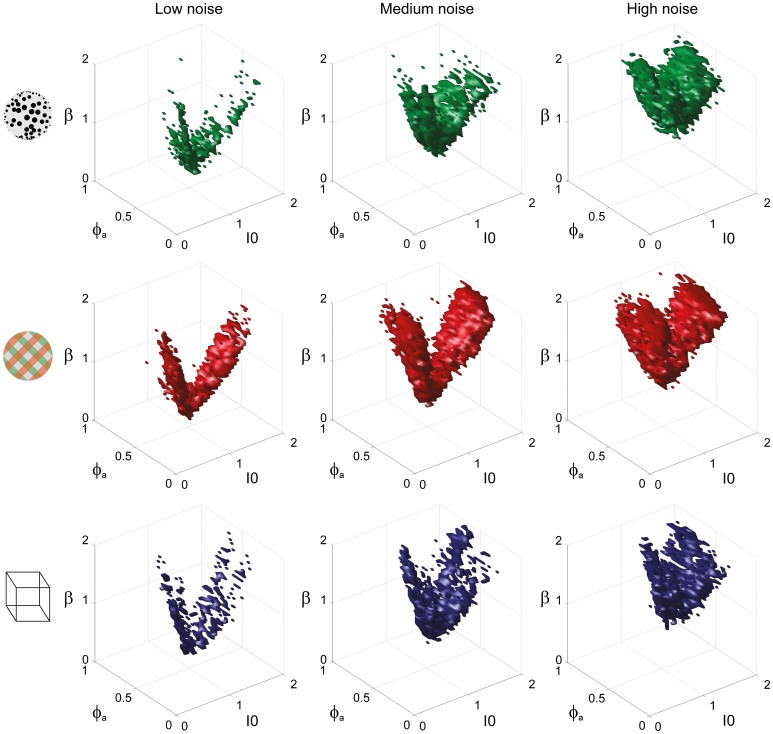
**Matching volumes depend on the assumed level of noise.** Union of matching volumes for all data sets from KD displays (top row), BR displays (middle row), and NC displays (bottom row). Assuming low noise (σ_n_ ∈ [0.01, 0.11]) displaced matching volumes to the margins of the bistable regime (left column), whereas an assumption of high noise (σ_*n*_ ∈[0.21, 00.35]) shifted matching volumes to the center of that regime (right column). Medium levels of noise (σ_*n*_ ∈ [0.13, 0.19]) produced the matching volumes shown in the middle column. The dependence of matching volumes on the assumed level of noise is also shown by the dashed contours in Figure 10.

To examine the “operating regime” of human observers in more detail, we carried out additional simulations in several two-dimensional subspaces, three of which are shown in Figure 10 (ϕ_*a*_ = 0.25, *I*_0_ = 0.5, and β = 1.75). These detailed simulations revealed that, depending on the assumed level of noise, human observers operate in different shell-like volumes of the bistable regime, each of which follows the bifurcation surface at some distance. As the assumed noise level increased from low (σ_*n*_ ∈ [0.01, 0.11]) to middle (σ_*n*_ ∈ [0.13, 0.19]) to high (σ_*n*_ ∈ [0.21, 00.35]), the distance to the bifurcation surface increased. Thus, the perceptual dynamics of most observers was matched by a shell-shaped volume at the margins of the *bistable* regime or, equivalently, near but not at the brink of the *oscillatory* regime (see also Figure 11).

### Shape and location of functional “sweet spot”

Is there a functional reason as to why multi-stable perception should operate in this particular regime? On the one hand, deep inside the *bistable* regime (strong β and weak ϕ_*a*_), perception is particularly stable (dominance times are particularly long). On the other hand, at the bifurcation boundary between the *oscillatory* and *bistable* regimes (β and ϕ_*a*_ proportional), perception is particularly sensitive to differential input (small imbalances between *I*_1_ and *I*_2_). Accordingly, any regime combining perceptual stability with perceptual sensitivity would constitute a functional “sweet spot.”

**Figure 12:**
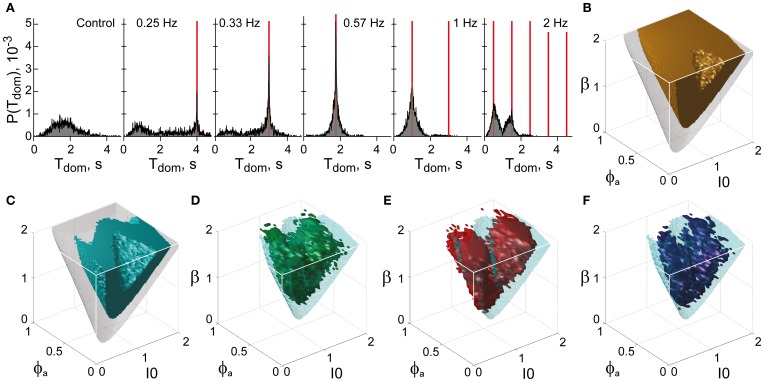
**Functional “sweet spot” combining perceptual stability and sensitivity. (A)** Frequency resonance driven by input modulation. Distribution of dominance times without modulation (far left) and for different modulations (red lines mark half-periods, from 0.25 to 2 Hz). A resonance peak is evident when the modulation half-period coincides with the peak of the unmodulated distribution. **(B)** Volume of maximal stability (orange, *T*_dom_ ≥ 1 s), compared to bistable regime (transparent gray). **(C)** Functional “sweet spot” combining maximal stability with maximal sensitivity to input fluctuations (cyan, frequency resonance measure *P*_1_ ≥ 1.2), compared to bistable regime (transparent gray). **(D–F)** Comparison of functional “sweet spot” (cyan) with regions matching perceptual dynamics of human observers for KD, BR, and NC displays (**D–F**, respectively).

To locate this “sweet spot” in terms of the LC-model, we computed the parameter volume providing exceptional stability (dominance periods >1 s, Figure 12B) and intersected it with the volume providing exceptional sensitivity (Figure 12C). To quantify sensitivity, we established frequency resonance *under the assumption of medium noise* (σ_*n*_ = 0.15). Frequency resonance is a sensitive method for probing the “operating point” of a dynamical system and is well established for the multi-stable perception of human observers (Kim et al., 2006).

Specifically, a periodic, anti-phase modulation of input strengths *I*_1, 2_ induces frequency resonance in the form of periodic reversals of dominance (Figure 12A). The input modulation moves the bifurcation boundary back and forth (with the movement range depending on modulation amplitude). Periodic reversals are triggered as soon as the boundary displacement reaches the “operating point” (i.e., the operative parameter combination) of the system under investigation. The system's sensitivity to input modulation may therefore be measured either in terms of modulation amplitude or, equivalently, in terms of the multiplicative increase of reversal probabilities around the resonance frequency (*P*_1_ measure, see “Materials and Methods”). The larger the *P*_1_-measure, the less modulation amplitude is needed to trigger a perceptual reversal.

The functional “sweet spot” of the LC-model, which combines maximal stability and sensitivity (*T*_dom_ > 1 s and *P*_1_ > 1.2), is illustrated in Figure 12C. It formed a shell-shaped volume which followed the bifurcation surface at a distance and was restricted to small values of adaptation. Remarkably, the volumes matching observer dynamics were largely coextensive with this “sweet spot” (Figures 12D–F). A more detailed comparison was possible in the planar subspaces of Figure 10, which juxtaposed the regions matching observer dynamics for low, medium and high noise (colored contours) and the functional “sweet spot” for medium noise (dotted contours). Note that it was the perceptual operating regime for *medium noise* (not for low or high noise) which best matched the functional “sweet spot” for medium noise.

## Discussion

We have compared the dynamics of multi-stable perception with a class of generative models in order to assess the effective contributions of competition, neural adaptation, and neural noise. Astonishingly, we find that highly heterogeneous measurements from different observers and displays consistently constrain these models to the same narrow operating regime (21 of 24 data sets). Moreover, this operating regime falls in a particularly interesting region from the point of view of perceptual performance. Specifically, it falls in a shell-shaped volume at some distance from the bifurcation boundary, which uniquely combines stability of perceptual outcome with sensitivity to input modulations. This constitutes compelling evidence that the temporal dynamics of perceptual inference is functionally optimized.

### A simplistic hypothesis

We have tested the hypothesis that different multi-stable phenomena reflect a common mechanism, namely, tectonic shifts of neural activity arising spontaneously within an attractor neural network that may well be distributed across distant cortical areas (Braun and Mattia, 2010). Presumably, a multi-stable display stimulates recurrent neural networks with several distinct steady states of neural activity (“attractor states”), which embody the cumulative residue of prior visual experience. These steady states are not absolutely stable, but are continually destabilized by neural adaptation and by neural noise. The result is an irregular, saltatory dynamics in which stable episodes are punctuated by rapid transitions.

The essential part of this hypothesis is the existence of a balance between competition, neural adaptation, and neural noise. Its precise mathematical formulation [here, the Laing and Chow model (Laing and Chow, 2002)] is only of secondary importance. Accordingly, we would expect that quantitatively different formulations of the same stabilizing and destabilizing factors should lead to qualitatively similar results. Consistent with this expectation, Shpiro et al. (2009) have shown that the broad “operating regimes” defined by the dominance distribution generalize over different models. It remains to be seen whether the same is true for the narrower “operating regimes” reported here (defined by both dominance distribution and history-dependence of multi-stable perception).

The hypothesis advanced here is admittedly simplistic in that it neglects many important aspects of multi-stable perception, such as its dependence on input strength (Moreno-Bote et al., 2007; Wilson, 2007; Seely and Chow, 2011) or its persistence across gaps in stimulation (Leopold et al., 2002; Maier et al., 2003; Brascamp et al., 2008; Pastukhov and Braun, 2008). Moreover, in treating multi-stable perception as a stochastic dynamical system, it ignores volitional processes such as attention shifts or eye movements.

There are two ways to justify this omission. Firstly, there is compelling evidence that reversals in the appearance of multi-stable displays do occur spontaneously, requiring neither attention nor eye movements (Lee et al., 2007; Pastukhov and Braun, 2007), except perhaps in some special situations (Zhang et al., 2011). Secondly, it seems likely that attention shifts and eye movements are part and parcel of the spontaneous dynamics we are postulating here. Recent evidence that reversals engage attentional mechanisms in a feedforward manner (Knapen et al., 2011) is consistent with the latter possibility.

In the end, we feel that the astonishing success of this simplistic hypothesis speaks for itself, especially as it extends to multi-stable displays (NC) known to be particularly susceptible to voluntary control (Meng and Tong, 2004).

### A hidden consistency

Our main finding is that the seemingly heterogeneous perceptual dynamics, which different observers exhibit with different multi-stable displays, conceals a hidden consistency. It has often been noted that the variability of dominance times is stereotypical, whereas mean dominance times are not (Murata et al., 2003; Brascamp et al., 2005; van Ee, 2005). On this basis, previous studies have concluded that human observers exhibit a bistable dynamics (Moreno-Bote et al., 2007), or that they operate in the vicinity (on either side) of the bifurcation separating bistable and oscillatory regimes (Shpiro et al., 2009). In contrast to these earlier studies, we also took into consideration the weak (but significant) dependence of dominance times on prior perceptual history (Pastukhov and Braun, 2011). These additional constraints revealed a consistent and narrow operating regime of human observers.

If multi-stable dynamics is so consistent, why do mean dominance times vary so widely between displays and observers? Our findings suggest at least a partial answer: when a dynamical system operates near a bifurcation, its evolution over time is not dominated by a single mechanism and parameter, but by a mixture of mechanisms and a combination of parameters. Indeed, for any given value of the time-constant τ_*a*_ of adaptation, small perturbations in the other parameters of the Laing and Chow model (Laing and Chow, 2002) generate considerable variance in the dominance time *T*_dom_ and, independently, in the time-constant τ_*H*_ of cumulative history. As a consequence, the pair-wise correlations between τ_*a*_, *T*_dom_ and τ_*H*_ are quite poor (Pastukhov and Braun, 2011).

### Near, not at, the brink

If our mechanistic hypothesis captures the essence of the situation, then visual perception operates in a marginally stable regime, near the brink of an oscillatory instability. According to the theory of dynamical systems, the Hopf bifurcation at the brink of an oscillatory instability constitutes a state of criticality (Camalet et al., 2000), in which signal processing is often found to be optimal in terms of sensitivity, dynamic range, or response latency. Several recent studies have shown that the dynamic range of the system response is enlarged (Kinouchi and Copelli, 2006), and the amount of information transferred increases (Beggs et al., 2003; Plenz and Thiagarajan, 2007; Shew et al., 2009), at the point of criticality. Indeed, operating at or near criticality may be a general principle of brain function (Bak, 1996).

The operating regime we have identified lies at some distance from the bifurcation boundary: it falls near, but not directly at, the brink of the oscillatory instability and is restricted to moderate strengths of adaptation. The functional advantage of such a *marginally stable* regime—in terms of relative stability of perceptual outcome and high sensitivity to input modulations (Figure 10)—may be understood as follows: Both dominance and response times are short at the bifurcation, but grow longer as the system enters more deeply into the bistable regime. A compromise—relatively long dominance and short response times—is reached at some distance to the bifurcation. When the input changes from being balanced (*I*_1_ = *I*_2_) to being biased (*I*_1_ < *I*_2_), the bifurcation border moves toward the bistable region. Accordingly, a system previously situated *near* the border may now find itself *at* the border and hence able to respond with a rapid reversal. In short, being *near*, but not directly *at*, the bifurcation affords both stability when the input is constant and sensitivity when the input changes.

### Stability vs. sensitivity

If visual inference is based on attractor dynamics (Braun and Mattia, 2010; Rolls and Deco, 2010), a goal conflict between stability and sensitivity seems unavoidable. Presumably, a stable and compelling appearance of a visual scene recruits numerous associations at all levels of visual processing—edges, surfaces, objects, generic context, episodic context. In terms of attractor dynamics, reciprocal excitation between visual and memory activity would be expected to stabilize a particular pattern of activity (and, thus, a particular appearance). The downside to this stabilization would be reduced sensitivity to incremental changes in the visual input, for attractor dynamics would tend to counteract any change and to restore the activity pattern that conforms to the activated memories. Accordingly, if the system is to remain sensitive to incremental input changes, associative stabilization by memory traces must not go too far. A combination of neural noise and neural adaptation would seem to offer an appropriate strategy for balancing stability and sensitivity, as this would also ensure that alternative interpretations are exhaustively explored.

### Exploitation-exploration dilemma

The present findings have important implications for theories of perceptual inference (Kersten et al., 2004). Given an exhaustive store of prior information, the outcome of Bayesian inference is deterministic. However, if the store of prior knowledge must be acquired by reinforcement learning (i.e., by trial and error), an inferential system faces the “exploitation-exploration dilemma” (Sutton and Barto, 1998). One the one hand, it must *exploit* what it knows already by following successful precedents from the past. On the other hand, if it is to expand its knowledge, it must *explore* alternative possibilities that may prove more successful in the future. The dilemma is that neither strategy can be pursued to the exclusion of the other. At the mechanistic level, such an inferential system must balance prior experience against current input. Favoring the former foregoes *exploring* novel inferences and compromises the *sensitivity* of inference (as input details are ignored). Favoring the latter foregoes the *exploitation* of prior knowledge and impairs the *stability* of inference (as input details are unduly amplified). Several authors have formulated similar thoughts in connection with perceptual inference (Hoyer and Hyvärinen, 2003; Hohwy et al., 2008; Sundareswara and Schrater, 2008; Moreno-Bote et al., 2010, 2011).

### Exception or rule?

Does marginal stability characterize only perfectly ambiguous, laboratory situations—such as the multi-stable displays investigated here—or does it apply also to real-world visual scenes? The answer hinges on whether the phenomenal appearance of real-world scenes is entirely stable, or whether it fluctuates in some way. Indeed, real-world objects evoke “contextual associations” such as, for example, episodic memories of prior personal experience, or generic knowledge about prototypical uses and locations (Bar, 2004, 2009b). The activation of such contextual associations is temporary and new associative possibilities are continuously being explored (Bar, 2009a). Contextual associations strongly color phenomenal appearance, presumably by activating perceptual representations in the manner of mental imagery (Moulton and Kosslyn, 2009). In certain impoverished visual displays—such as two-tone faces or Rorschach ink blots (Mooney et al., 1957)—this influence is particularly evident. Accordingly, we speculate that multi-stable phenomena form a continuum, ranging from perfectly ambiguous situations (such as the canonical multi-stable displays studied here), to partially ambiguous images with multiple readings of different plausibility (such as two-tone faces), to real-world images with a large number of subtly different associations.

### Final thoughts

We propose a functional hypothesis as to why visual perception is marginally stable in general, and marginally multi-stable in ambiguous situations. Specifically, we propose that vision operates in a dynamical regime that uniquely combines stability and sensitivity, thus optimizing performance. At the mechanistic level, we speculate that this balance may be struck by attractor dynamics encompassing both visual and memory representations.

### Conflict of interest statement

The authors declare that the research was conducted in the absence of any commercial or financial relationships that could be construed as a potential conflict of interest.
